# Annual Report of the Provost Marshal General, from November 1863 to November 1864

**Published:** 1865-06

**Authors:** 


### BOOKS REVIEWED.

*Annual Report of the Provost Marshal General, from November* 1863 *to November* 1864.

We have received from Surgeon J. H. Baxter, U. S. V., Chief Medical Officer of the Provost Marshal General's Bureau, a copy of the above Report, a pamphlet of about fifty pages. Of the portion of it devoted to the business proper of the Bureau, we of course have nothing to say, but the medical portion of it, by far the greater part, must be interesting to all medical men who are interested in vital statistics. The statistics embraced are those of the draft of 1863, (known as the *first* draft,) and that of March 14, 1864, (second draft,) the draft of July 18, 1864, and the fourth of 1865, will be embraced, we suppose, in a subsequent report.

A most difficult problem to be solved in carrying out the provisions of a conscription act is to decide upon a list of diseases which shall constitute causes of exemption. To be sure the progress of this war has so far educated every medical man that he must have a tolerably correct idea of what would and what would not disqualify a man for the duties of a soldier in the field; but, clearly, it would be impracticable to leave the matter to individual discretion, and to frame such a list as shall neither furnish too easy a means of escape, nor prove too exacting upon those really incapable to do duty, requires no small tact. This difficulty has made itself manifest in the fact that three lists of disqualifying infirmities have been issued since the act went into effect, and it is worthy of notice that each list has been more stringent than its predecessor. The reason for this change, though obvious enough to any one who has had any experience in the workings of the conscription, has not been appreciated by the mass of the people, and no little grumbling has resulted. In the outset, both the authorities and the people, were inclined to place the examination of a drafted man very nearly on a par with that of a recruit, neither realizing how few men comparatively, are physically perfect. The recruit is to receive a bounty, and Government demands that he shall be fit for any duty, but the drafted man is already in the service, and if not fit for severe duty, he may be put into less severe situations when he will be just as valuable as a better man. That is, in the one case it is a question whether a man shall be taken who is offered, in the other whether a man shall be retained who is already taken. In time of peace the two standards could properly be made more nearly alike, but in a war, and especially such a war as has just closed, good sense dictates such a policy as has been pursued, and patriotism requires that people should acquiesce in it.

The effect of this change of causes for exemption is seen in the fact stated in this Report that whereas, in 1863, the number of exemptions was in the ratio of 314.02 to 1000 examined, in 1864 the ratio had fallen to 257.02 to 1000; nor can this decrease be ascribed to the population having been in any degree sifted out by the examination of 1863, for, owing to the change in the list of causes the exemptions for physical causes of 1863, did not hold good in 1864.

We should be glad to copy the list of disqualifying infirmities, and make it the basis of some remarks, but our space forbids; however, we can in the main endorse the following from Surgeon Baxter's Report:

"I do not recommend, at present, any change in the list of diseases and infirmities governing boards of enrollment in the exemption of drafted men; believing that, though faults may exist, yet with a proper construction and understanding of the list, as now given in paragraph 85, Revised Regulations Provost Marshal General's Bureau, all drafted men, who are really unfit for military service can be exempted in accordance with its provisions."

A large portion of the pamphlet is occupied with a very thorough tabulation of the results of the examinations under the two drafts included in the Report. We had intended giving an analysis of these statistics, which are both valuable and interesting, but refrain from doing so at this time in the hope that the final reports will ere long enable us to reason from a more extended basis. We are the more willing to adopt this course, since the forms adopted for the forthcoming reports promise a more thorough analysis of the examination, and more copious statistical information. One or two considerations, however, which might become sources of error in a superficial examination of the tables here given, we cannot refrain from noticing. One is, that very many drafted men feeling themselves to be fit for duty, have waived the privilege of examination and furnished substitutes, consequently their names do not appear on the Surgeon's books. Had these all been included the result would have been undoubtedly somewhat modified. The other refers to the comparison made between the results of examinations here and in Europe, whereby it appears that the percentage of rejections is much smaller in this country. It should be borne in mind that the European examinations were not made under such a pressing necessity as has governed here, and, consequently exemptions and rejections have been made for merely technical defects which, under the present regulations, do not constitute a sufficient cause for discharge. We have ourselves seen men of European birth who held certificates of exemption from conscription[1] given them there who could not be exempted here, though the same cause existed still. A comparison between the recruits rejected here and abroad would, we think, show a less difference.

By far the most interesting tables are those containing the average age, heighth and chest measurements of men examined, but of those we purpose to speak hereafter, only pausing now to express our regret that *weight* was not also made a subject of report, as we believe, within certain limits, that is a valuable indication of physical condition.

The intelligent industry manifested in the preparation of this document merits great praise, and entitles Surgeon Baxter to the especial thanks of all who are interested in vital statistics. The lessons to be learned from these, and the corresponding tables which will eventually be prepared from our voluminous hospital records, will be of universal value, and will constitute the grandest addition ever made to the science of military medicine, surgery and hygiene.

In conclusion, we are glad for the honor of our profession, to copy the following paragraph from the Report, and to endorse it so far as our knowledge extends, notwithstanding many petty calumnies which have been indulged on the subject. We quote, "and from personal inspections and reports of competent medical inspecting officers, I feel justified in stating that surgeons of boards of enrolment, as a class, are gentlemen of education and ability, reliable and honest, and there is every reason to believe, are influenced in the performance of the responsible duties pertaining to their position, rather from a desire to serve their country than the meagre compensation received for their services." P.

